# COVID-19 Contact Tracing as an Indicator for Evaluating a Pandemic Situation: Simulation Study

**DOI:** 10.2196/43836

**Published:** 2023-04-06

**Authors:** Manuel Marques-Cruz, Diogo Nogueira-Leite, João Miguel Alves, Francisco Fernandes, José Miguel Fernandes, Miguel Ângelo Almeida, Patrícia Cunha Correia, Paula Perestrelo, Ricardo Cruz-Correia, Pedro Pita Barros

**Affiliations:** 1 Department of Community Medicine, Information and Decision in Health Faculty of Medicine University of Porto Porto Portugal; 2 Center for Health Technology and Services Research Faculty of Medicine University of Porto Porto Portugal; 3 NOVA Health Economics & Management Knowledge Center NOVA School of Business and Economics NOVA University of Lisbon Lisboa Portugal; 4 Public Health Unit Marão e Douro Norte Agrupamento de Centros de Saúde Marão e Douro Norte Administração Regional de Saúde do Norte Vila Real Portugal; 5 Public Health Unit Baixo Mondego Agrupamento de Centros de Saúde Baixo Mondego Administração Regional de Saúde do Centro Figueira da Foz Portugal; 6 Public Health Unit Alto Ave Agrupamento de Centos de Saúde Alto Ave Administração Regional de Saúde do Norte Fafe Portugal; 7 Public Health Unit Póvoa de Varzim Agrupamento de Centros de Saúde Póvoa de Varzim - Vila do Conde Administração Regional de Saúde do Norte Vila do Conde Portugal; 8 Public Health Unit of Local Health Unit of Guarda Unidade Local de Saúde da Guarda Guarda Portugal

**Keywords:** COVID-19, public health, public health surveillance, quarantine, infection transmission, epidemiological models

## Abstract

**Background:**

Contact tracing is a fundamental intervention in public health. When systematically applied, it enables the breaking of chains of transmission, which is important for controlling COVID-19 transmission. In theoretically perfect contact tracing, all new cases should occur among quarantined individuals, and an epidemic should vanish. However, the availability of resources influences the capacity to perform contact tracing. Therefore, it is necessary to estimate its effectiveness threshold. We propose that this effectiveness threshold may be indirectly estimated using the ratio of COVID-19 cases arising from quarantined high-risk contacts, where higher ratios indicate better control and, under a threshold, contact tracing may fail and other restrictions become necessary.

**Objective:**

This study assessed the ratio of COVID-19 cases in high-risk contacts quarantined through contact tracing and its potential use as an ancillary pandemic control indicator.

**Methods:**

We built a 6-compartment epidemiological model to emulate COVID-19 infection flow according to publicly available data from Portuguese authorities. Our model extended the usual susceptible-exposed-infected-recovered model by adding a compartment Q with individuals in mandated quarantine who could develop infection or return to the susceptible pool and a compartment P with individuals protected from infection because of vaccination. To model infection dynamics, data on SARS-CoV-2 infection risk (IR), time until infection, and vaccine efficacy were collected. Estimation was needed for vaccine data to reflect the timing of inoculation and booster efficacy. In total, 2 simulations were built: one adjusting for the presence and absence of variants or vaccination and another maximizing IR in quarantined individuals. Both simulations were based on a set of 100 unique parameterizations. The daily ratio of infected cases arising from high-risk contacts (*q* estimate) was calculated. A theoretical effectiveness threshold of contact tracing was defined for 14-day average *q* estimates based on the classification of COVID-19 daily cases according to the pandemic phases and was compared with the timing of population lockdowns in Portugal. A sensitivity analysis was performed to understand the relationship between different parameter values and the threshold obtained.

**Results:**

An inverse relationship was found between the *q* estimate and daily cases in both simulations (correlations >0.70). The theoretical effectiveness thresholds for both simulations attained an alert phase positive predictive value of >70% and could have anticipated the need for additional measures in at least 4 days for the second and fourth lockdowns. Sensitivity analysis showed that only the IR and booster dose efficacy at inoculation significantly affected the *q* estimates.

**Conclusions:**

We demonstrated the impact of applying an effectiveness threshold for contact tracing on decision-making. Although only theoretical thresholds could be provided, their relationship with the number of confirmed cases and the prediction of pandemic phases shows the role as an indirect indicator of the efficacy of contact tracing.

## Introduction

### Background

Contact tracing is a fundamental activity in public health and is the process of identifying, triaging, and monitoring individuals exposed to a communicable disease to prevent secondary transmission [1]. When systematically applied, contact tracing leads to the breaking of transmission chains, and its valuable role in controlling COVID-19 transmission is widely recognized in the context of low transmission and active community transmission [2]. It is also acknowledged that the earlier the identification of infection cases and individuals to trace, the higher the likelihood of the pandemic situation being controlled even in instances where contact identification is incomplete [1,3,4].

In Portugal, contact tracing and the imposing of quarantine measures are tasks specific to public health units [5]. The capacity of local public health units to perform contact tracing is constrained by the availability of technological and workforce resources [3]. Several simulation studies have demonstrated that contact tracing could only be effective if a combination of high adherence to quarantine measures, minor delays from symptom onset to isolation of cases, and an increased number of contacts traced occurred [3,6-8].

Such conditions were more likely to be verified in moments with a lower number of confirmed cases (ie, corresponding to the troughs of the epidemic curve) [9]. Moreover, the initial Portuguese guidelines for tackling the pandemic predicted that contact tracing efforts would be abandoned when the pandemic entered a community transmission phase [10]. Therefore, it should be deemed necessary to estimate a threshold beyond which contact tracing and potential individual quarantine measures cease to be effective in breaking infection chains and, consequently, diminishing SARS-CoV-2 infection rates in the community [11]. This information may be used as a decision-making support tool for imposing generalized population containment measures and offers important lessons concerning population control of viral infections with characteristics similar to those of SARS-CoV-2 [12].

The effectiveness of contact tracing as a mechanism for breaking transmission chains occurs because of the quarantine of contacts of confirmed infected cases. Should these contacts develop the disease, they will not transmit it to other community members [1]. Thus, the hypothetical identification of all contacts of all SARS-CoV-2–infected individuals would necessarily lead to the breaking of all transmission chains. In other words, in a scenario where all the contacts of all infection cases were identified, new infection cases would only occur in those contacts. Hence, the effectiveness threshold of a contact tracing and quarantine strategy could be derived from the proportion of infection cases that arise in contacts of confirmed cases.

Hence, the effectiveness threshold of contact tracing is the point at which its utility as a health intervention that includes quarantine in controlling and breaking transmission chains is defined. The proportions of confirmed cases from quarantined individuals above this threshold could be indicative of effective contact tracing, and the pandemic combat strategy could rely mainly on this intervention. In contrast, proportions below the threshold could indicate that contact tracing is not effective and that further interventions may be necessary to stop transmission.

The proportions of confirmed cases from quarantined individuals lower than the effectiveness threshold may have occurred at different stages throughout the pandemic, mainly in periods of case surges, situations in which there was a need to implement more restrictive measures (namely, general or selective confinements). Imposing confinements has been demonstrated to affect pandemic control regarding case numbers, hospital admissions, and deaths because of COVID-19 when implemented at least 14 days before the peak of a case surge [13,14]. In Portugal, the decision to impose a population lockdown was primarily based on the 14-day incidence rate; the transmissibility rate (Rt); and, more recently, the critical care bed occupancy rate [15].

Despite the public availability of several global databases on COVID-19, especially concerning the number of new cases, deaths, tests performed, and vaccination data, there is no information on the number of infected cases coming from individuals identified as high-risk contacts through contact tracing. Furthermore, data on quarantined individuals are scarce. Portuguese data were until recently an exception in that the Directorate-General of Health (DGS) reported in a daily bulletin on COVID-19 the number of high-risk contacts identified, defined as individuals in quarantine by mandate from health authorities [16]. The Data Science for Social Good (DSSG) initiative developed a data repository [17] for COVID-19 in which it gathered, compiled, and curated the data made public by the DGS in these bulletins.

Infection dynamics and the effectiveness of contact tracing are not only influenced by the vaccinated population (for which the DSSG also kept curated data). They may also be affected by the prevalence of different SARS-CoV-2 variants at different points in time. The European Centre for Disease Prevention and Control keeps a data repository on the prevalence of different variants in Europe reported through The European Surveillance System in an open-access database [18].

### Objectives

The aim of this study was to identify the ratio of COVID-19 cases that occurred in individuals in quarantine mandated by health authorities and the potential use of this ratio as a proof of concept of an indicator for assessing pandemic control in parallel with other established criteria such as the transmissibility index (Rt) and incidence.

## Methods

### Study Design

In this study, we collected data on COVID-19 for Portugal and built an expanded structure to a susceptible-exposed-infected -recovered (SEIR) compartmental model to emulate the pandemic. All compartment data came from collected data except for those of protected individuals. The purpose of the model was to estimate the daily number of quarantined individuals who became infected and the daily number of susceptible and vaccinated individuals who were quarantined as data regarding those values were lacking.

We input different values for each known strain of SARS-CoV-2 (infection risk [IR] and time until infection and until the end of quarantine) and immunity from vaccination and ran the model for 690 days. Consequently, we estimated the daily ratio of cases arising from daily quarantined individuals (*q* estimate) using the number of daily confirmed cases as the denominator. We measured the correlation between this *q* estimate and data from confirmed cases.

According to the epidemic case curve, we defined 3 pandemic phases in the Portuguese data: interpandemic phase, alert phase, and pandemic phase. As a proof of concept, we estimated the best hypothetical cutoff for our *q* estimates to distinguish the interpandemic and alert phases and compared that theoretical cutoff with the timing of population confinement measures. Finally, we performed a 2-part sensitivity analysis. Initially, we ran a multiple linear regression on the thresholds that each set of parameters conveyed to assess how each parameter would change the threshold value. In addition, we fixed the maximum and minimum values for each parameter and measured the correlation with the main simulation results and threshold values obtained by changing all other parameters.

Table 1 includes all model inputs, values, and sources of each input. We also describe the only 2 outputs of the model, namely, the number of daily quarantined individuals who develop infection and the theoretical *q* estimate.

The compartment transition dynamics were in accordance with the following equations (note that only equations 1 and 3 were estimated, corresponding to compartments S and E, for which real data were not available. All other compartment data were directly collected from official reports [17]):

S' = κQ+ ρP − (φ + ψ + ε)S **(1)**

Q' = φS+ χP − (γ + κ + ς)Q **(2)**

E' = I − γQ **(3)**

I' = γQ+ ιE − πI **(4)**

*R* = πI **(5)**

**Table 1:** Data sources and model inputs and outputs.

Parameter	Value	Source
Susceptible (S)	Base case: 10 million	Estimated
Protected through vaccination (P)	P (Protection) × V	Estimated
Quarantined (Q)	Base case: 0	DSSG^a^ [17]
Exposed not traced (E)	Base case: 0	Estimated
Infected (I)	Base case: 2	DSSG [17]
Recovered from infection (R)	Base case: 0	DSSG [17]
Vaccinated (V)	Base case: 0	DSSG [17]
Inoculation vaccine efficacy (Ve_0_)	10%-60% (uniform distribution)	Hall et al [19] and Polack et al [20]
Maximum vaccine efficacy (Ve_max_)	Ve_0_-95% (uniform distribution)	Hall et al [19] and Polack et al [20]
Waned vaccine efficacy (Ve_waned_)	(Ve_0_ + Ve_max_)/2	Estimated
Inoculation booster dose efficacy (Be_0_)	Ve_waned_-80%	Estimated
Maximum booster dose efficacy (Be_max_)	Be_0_-95%	Estimated
Waned booster dose efficacy (Be_waned_)	(Be_0_ + Be_max_)/2	Estimated
Time until maximum efficacy (∆te_max_)	15 d	Hall et al [19] and Polack et al [20]
Time until waned efficacy (∆te_waned_)	180 d	Hall et al [19]
Variant prevalence	Base case: other=100%; alpha, beta, gamma, delta, and omicron=0%	ECDC^b^ [18]
Maximum time until γ (∆tγ_max_)	2-14 days (uniform distribution)	DGS^c^ [5,21] and Wu et al [22]
Average time until γ (∆tγ_mean_)	2-∆tγ_max_ days (uniform distribution)	DGS [5,21] and Wu et al [22]
Maximum time until κ and ς (∆tκς_max_)	2-14 days (uniform distribution)	Estimated
Average time until κ and ς (∆tκς_mean_)	2-∆tκς_max_ days (uniform distribution)	Estimated
IR^d^ simulation A	10%-50% (uniform distribution)	ECDC [1], Karumanagoundar et al [23], and Tang et al [24]
IR simulation B	0.1%-2.5%	Calibration
Quarantined infected	γQ	Estimated
Ratio of cases from quarantined (*q*)	γQ/(γQ + ιE)	Estimated

^a^DSSG: Data Science for Social Good.

^b^ECDC: European Centre for Disease Prevention and Control.

^c^DGS: Directorate-General of Health.

^d^IR: infection risk.

### Data Collection

The sources of data are described in full in Table 1. The primary data source to meet this study’s aim was the DSSG COVID-19 data repository [17]. The data included the number of confirmed cases, daily new cases, people under surveillance, people fully vaccinated, and individuals with vaccine booster doses (using any of the vaccines available in Portugal) between the first confirmed case of COVID-19 in Portugal (March 2, 2020) and January 20, 2022. Open-access data from The European Surveillance System regarding the weekly prevalence of SARS-CoV-2 variants were also collected through the European Centre for Disease Prevention and Control open repository [18] for the same period. In the absence of specific data on the number of cases of COVID-19 among high-risk contacts, we built a simulation model to calculate the ratio of daily cases from those contacts using the available data.

### Epidemiological Compartmental Model

Data were inserted into an epidemiological model based on compartmental models already applied to COVID-19 and other epidemiological contexts [25,26]. Several expanded models to an SEIR model have been attempted, including either one or more quarantine compartments and one or more protected individuals through vaccination [27]. In addition, the concept of high-risk exposure and exposed individuals (frequently included in compartment E) did not entirely comprise the DGS definition of high-risk contact in that, in all models, an exposed individual could not return to being susceptible [28]. Furthermore, in most models, a quarantined individual could have come from being susceptible and either return to being susceptible or progress to being infected [29]. Other quarantine definitions, different from the idea of prophylactic isolation, came from infected individuals [30].

**Figure 1:**
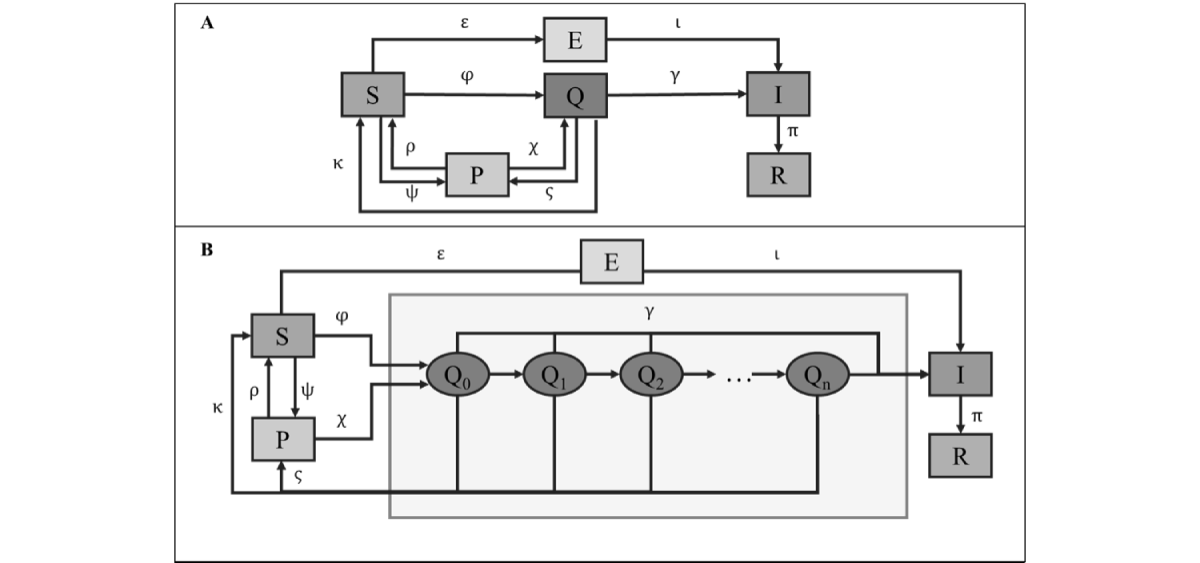
Compartmental model without subcompartments (A) and with subcompartments (B). E: individuals exposed to SARS-CoV-2 who will develop COVID-19 and have not been traced by health authorities; I: infected individuals; P: subset of susceptible individuals protected from SARS-CoV-2 infection through vaccination; Q: quarantined individuals; Q0, Q1, Q2,..., Qn: subcompartments of the quarantine compartment (each number represents the number of days since exposure); R: recovered individuals; S: susceptible individuals.

Moreover, 2 transitions have not been reported in the reviewed literature on expanded SEIR models, namely, the direct transition between compartments S and I and the transition between vaccinated or protected compartments and quarantine or exposure compartments. As our model needed to comprise these transitions, several differences from already published models had to be introduced. To keep with the base SEIR structure, compartment E included only individuals who would progress to compartment I without previous contact tracing. The model was extended with compartments Q and P. Figure 1 illustrates the compartmental model used and the transitions between compartments and subcompartments.

Compartment S (susceptible) is the initial compartment of the model (ie, the starting point for all individuals). From compartment S, individuals can progress to compartments P, Q, and E.

Compartment P (protected) refers to the group of individuals who are immune to SARS-CoV-2 infection because of vaccination. Although the main effect of vaccines is protection against severe disease and not protection against infection [20], short-term efficacy in infection prevention has been proven [19]. This preventive effect is considerably reduced 6 months after the date of inoculation. When classified as high-risk contacts through contact tracing, individuals in this compartment could be quarantined regardless of their vaccination status in line with Portuguese norms that only lifted these compulsory measures for fully vaccinated individuals on January 10, 2022 [5].

Compartment Q (quarantined or exposed with tracing) includes all high-risk contacts of SARS-CoV-2–infected individuals for whom quarantine was mandated by a public health authority according to norms and guidelines [5] and the definition by the Portuguese DGS as “individuals under surveillance” applicable at each moment. Individuals in this compartment could return to compartment S. This compartment is further divided into subcompartments, each representing a day in which an individual stayed in the exposed compartment. Thus, the transitions φ and κ, χ and ς, and γ are the pooled transitions from each subcompartment inside compartment Q and compartments S, P, and I, respectively.

Compartment E (exposed without tracing) includes exposed individuals who will develop COVID-19 and have not been traced by health authorities. Compartment I consists of all the confirmed cases of SARS-CoV-2 infection.

Compartment R (recovered) includes all individuals who recovered from infection. Compartment R is the terminal compartment as the model is not circular. This is because an individual previously infected with COVID-19 cannot be considered a high-risk contact before 180 days have passed from the date of infection or when not presenting symptoms suggesting SARS-CoV-2 infection [21]. In other words, until the natural immunity gained from infection fades, there can be no transition between the recovered (R) and exposed (E) or quarantined (Q) compartments. When such immunity wanes, the individual should return to compartment S. However, and assuming a large enough number of individuals in compartment S (equal to or greater than the total number of confirmed cases in the analysis period), there is no need to consider transitions between compartments R and S—even in case of reinfection, each new case of COVID-19 can be regarded as a case from a different individual.

The daily ratio of cases arising from individuals in quarantine mandated by health authorities was given by the quotient of the daily transitions between compartments Q and I (represented by γ) and the daily total confirmed cases.

### Parameterization and Transitions

The compartmental model was run for 690 days (from March 2, 2020, to January 20, 2022). The data collected populated compartments Q, I, and R daily. In the absence of data for compartment S, an initial value of 10 million individuals was assumed, which is a frequently used approximation for the Portuguese population. This value ensured that the model was not circular and obviated the need to consider the possibility of reinfection. Data on compartments P and E were calculated, and expected values for other parameters were defined within each simulation run (Table 1).

Regarding daily transitions between compartments and subcompartments, the model was built in 3 steps: transitions from subcompartments inside Q (step 1), S-E-I transitions (step 2), and S-Q and P-Q transitions (step 3). Despite the existence of data for all compartments and some model parameters, owing to a lack of data in each of these steps regarding the actual number of individuals that transition in each iteration of the model, some parameters were input by defining expected values for the parameters that govern those transitions. Only those parameters were estimated (Table 1).

In step 1, Q-S, Q-P, and Q-I transitions are the sums of the number of individuals who each day (represented by each subcompartment) transition to compartments S, P, or I, respectively. Each day, transitions from each subcompartment are governed by the probabilities of staying in compartment Q (transition to the following subcompartment Q_i+1_), making the transition γ to compartment I, or returning to compartment S or P (transitions κ and ς). The probabilities of transition of each subcompartment were defined according to the IR of a high-risk contact of a confirmed COVID-19 case and to the mean and maximum periods an individual remains exposed until the person either develops the infection or is considered susceptible again. Any of these 5 parameters can vary with the viral variant to which the individuals were exposed and with the quarantine period. Therefore, in the context of the described model, the probability of transitioning from any subcompartment to compartment S, P, or I is given by the sum of the product of the transition probability of each specific variant and the number of individuals exposed to that variant. Step 1 is concluded after calculating the ratio of new daily cases of infection that came from compartment Q (resulting from transition γ) to the total number of new daily cases (*q* estimate).

Steps 2 and 3 aim to keep the model closed (a necessary condition for using compartmental models), allowing the model to simulate the following day. The number of individuals that follow the ε and ι transitions in step 2 is given by the difference between the number of new daily infections and the number of individuals in transition γ. The φ and χ transitions in step 3 are calculated as the difference between the number of exposed individuals (high-risk contacts) on the following simulation day and the number of individuals inside compartment Q after steps 1 and 2. The value of this transition corresponds to the number of individuals inside subcompartment Q_0_ at the beginning of step 1 on the following simulation day.

Finally, compartment P was defined according to 5 parameters, namely, vaccine efficacy after inoculation, maximum efficacy, and efficacy after a waning period as well as the elapsed time between vaccination and maximum efficacy and between maximum efficacy and waned efficacy. This compartment was calculated as the product of the probability of each vaccinated individual being protected from SARS-CoV-2 infection and the number of vaccinated individuals.

### Assumptions of the Simulation

To operationalize the proposed model, several assumptions were made, namely regarding the transitions involving compartments Q and P.

A Markov chain Monte Carlo simulation defined the transitions between the Q subcompartments. Consequently, to compute the transitions between subcompartments, a series of steps were taken (Textbox 1).

Regarding compartment P, and particularly vaccine efficacy, a constant rate was assumed between two events: (1) efficacy after inoculation and maximum efficacy and (2) maximum efficacy and waned efficacy. Hence, vaccinated individuals were probabilistically placed in compartment P each day according to the time elapsed since inoculation. After defining new values for booster efficacy, the same procedure was replicated for individuals with booster doses. Individuals in compartment P could transition to compartment Q, but their IR was set at 0. Individuals in compartments S and P would transition to compartment Q according to the ratio between those compartments.

Owing to the inherent variability in the timing of contact tracing and imposing quarantine, it is impossible to accurately define certain parameters in the model for each variant of SARS-CoV-2 and each vaccine. Therefore, we decided to simulate a set of different parameterizations for these parameters. For each variant, the maximum time elapsed for the transition to compartments I and S was assumed to range from 2 to 14 days. The mean time for these transitions ranged from 2 days to the maximum time previously set for each variant. IR was set to range from 10% to 50% [1,23,24], and we considered 6 different variants, namely, “alpha,” “beta,” “gamma,” “delta,” “omicron,” and “others” (according to collected data). We assumed that the weekly prevalence of each variant [18] was equal to the prevalence on each day of that week, and individuals in the φ transition would be distributed to each variant according to that prevalence.

Regarding vaccination, we defined an efficacy between 10% and 60% after inoculation and a maximum efficacy of up to 95% [19,20]. We assumed a waned efficacy as the mean value between both efficacies. Booster vaccines had an assumed efficacy between the waned efficacy previously defined and 80% after inoculation and a maximum efficacy of up to 95%. A period of 15 days until maximum vaccine efficacy, and a period of 180 days until waned efficacy were assumed [19,20]. Individuals from compartment P entered the model as exposed to variant “others” with an IR set at 0%—in other words, they were set to follow the Poisson distribution estimated for returning to compartments S and P for the variant “others.”

**Steps taken to compute the transitions between subcompartments.**

- To calculate the different transition probabilities, we assumed that everyone who transitions to compartment Q has a predetermined probability of becoming infected, called infection risk (IR). IR is specific to each variant.
- For each variant included, 2 different Poisson distributions were applied to the individuals who would become infected and those who would return to compartments S and P. Each distribution had a maximum and mean time until transition.
- From the combined transition probabilities of each distribution and according to the IR, a transition table was constructed, including the pooled transition probabilities from each subcompartment to the following subcompartment, to compartments S and P, and to compartment I.
- A Monte Carlo simulation was then applied to this transition table. Therefore, on each simulation day, an individual in a subcompartment of the Q compartment transitions probabilistically to the following subcompartment, to compartments S and P, or to compartment I.

### Description of the Simulations

In total, 2 different simulations were performed, approaching the objective in 2 different ways. Table 2 summarizes the main characteristics and differences between the simulations. We first defined that each simulation should have a run time of ≤9 hours. An iteration of the model was tested on a computer with an Intel64 Family 6 Model 126 Stepping 5 GenuineIntel processor with a maximum clock frequency of 1498.0 MHz with 4 physical cores, 8 logical cores, and 15.60 GB of RAM using the operating system Windows 10.0.19041. With this hardware and software combination, the response time (time taken from start to end of the process) for this iteration was 10 seconds [31].

Simulation A aimed to be as approximate as possible to what we could expect in real life by accounting for the potential influence of variants and vaccination. However, it assumed that the risk of infection was fixed for all variants at the beginning of the simulation. For this simulation, 100 parameterizations were established, and these were quadrupled to reflect the presence and absence of the influence of variants and vaccination. Each parameterization was iterated 100 times, totaling 40,000 model iterations with an estimated run time of 8 hours and 20 minutes.

Simulation B ignored the presence of different variants (we assumed that all cases came from the variant “others”) and estimated the maximum value of IR for which the model kept the *q* estimate at <1. However, as in simulation A, the risk of infection in exposed individuals was fixed for each parameterization and did not fluctuate throughout each iteration. This simulation used the same set of 100 parameters used in simulation A, doubled to account for the presence or absence of vaccination. The IR started at 100% with each iteration interrupted, and this value was reset and diminished by 0.1 percentage points every time the daily *q* estimate was >1. We assumed that this approach might lead, in extremis, to circa 1000 iterations of each parameterization. Although mostly incomplete because of the described procedure to calibrate the IR, this number of iterations would lead model B to take 20 times longer to run than model A. Therefore, the research team opted to conduct only 10 iterations of each parameterization, which, jointly with half of the total parameterizations of simulation A, kept the estimated simulation running time at 8 hours and 20 minutes.

**Table 2:** Main characteristics of the implemented simulations.

	Simulation A	Simulation B
Used information on vaccines	Yes	Yes
Used information on variants	Yes	No
Number of different parameterizations, n	400 (100 × 4)	200 (100 × 2)
Ceiling value for the ratio of cases (*q* estimate)	No	Yes
Parameters included fixed infection risk	Yes	Yes
Number of iterations per parameterization, n	100	Approximately 1000
Total model iterations, n	40,000	200,000
Estimated run time	8 h and 20 min	8 h and 20 min

### Data Analysis

The parameters’ characteristics were aggregated in means and medians and described for each simulation. For each simulation, the distribution of the ratio of daily cases resulting from the γ transition (*q* estimate) for each parameterization was combined into a single distribution according to the methodology implemented by Hill [32]. We also combined the results of each simulation into a single distribution according to the presence or absence of both vaccination and different variants in the model when applicable. Although the *q* estimate intended to represent the proportion of cases arising from high-risk contacts, certain parameter combinations yielded values of the *q* estimate of >1 (Textbox 2).

Since other COVID-19 measures for lockdown were presented in a 14-day moving average, we applied a 14-day moving average to the daily *q* estimate for each simulation and to the gathered data on daily cases. The Spearman correlation was calculated between the 14-day *q* estimate and the 14-day average number of cases.

Local maxima for 14-day data on confirmed infected cases were computed, and local minima immediately before and after the computed local maxima were maintained. By using an adaptation of the method used by Vázquez-Seisdedos et al [33] and applied by Gianquintieri et al [34] to data on COVID-19 cases, we determined the inflection point of the case curve that occurs between the local minimum and the local maximum. This inflection point corresponds to the beginning of a pandemic phase. Each pandemic phase was defined, in the context of this study, as the interval between an inflection point and the following local minimum, which had a local maximum between them.

It was assumed that, by definition, a contact tracing effectiveness threshold would have to be surpassed during a pandemic phase. Specifically, the value of the effectiveness threshold would have to occur in an alert phase, defined as the critical period between the wave’s inflection point and up until 14 days before the day when the peak number of cases was registered. In addition, it was assumed that contact tracing as a main strategy for pandemic combat was reset after the end of each pandemic phase (ie, local minimum after case peak) and the model entered an interpandemic phase. For this reason, an effectiveness threshold was calculated considering the interpandemic and alert phases. In other words, we included the entire period analyzed except for periods between the end of an alert phase and the end of each pandemic phase. The data between a local minimum and the next inflection point (interpandemic phase) were considered as controls for determining the threshold. As there was a more considerable period of control data than the period in the alert phase, we oversampled the test data. This classification into interpandemic, alert, and pandemic phases was loosely based on the World Health Organization pandemic phases for influenza [35].

This threshold estimation from the *q* estimates was a theoretical demonstration of the impact of applying the same procedure to the actual proportion of cases arising from high-risk contacts in anticipation of the need for populational lockdown measures that were ultimately imposed.

Effectiveness threshold values were estimated for each simulation using a receiver operating characteristic model applying the Youden method. The sensitivity, specificity, and positive predictive value (PPV) were calculated for the theoretical effectiveness threshold. Sensitivity represented the proportion of days in the alert phase in which the *q* estimate was below the effectiveness threshold. Specificity represented the proportion of days in the interpandemic phase in which the *q* estimate was above the effectiveness threshold. The PPV was the quotient of the number of alert phase days in which the *q* estimate was below the effectiveness threshold and the number of days in which the *q* estimate was below the effectiveness threshold.

Finally, we implemented a 2-part sensitivity analysis. First, we estimated a hypothetical threshold for each of the different parameterizations used (both in simulations A and B) and ran a multiple linear regression with the estimated thresholds as the dependent variable. We included 10% increases in IR of a high-risk contact of a confirmed COVID-19 case and mean and maximum periods during which an individual remains quarantined until they either develop the infection or are considered susceptible in days, for each strain, and 10% increases in maximum and waned efficacies for complete vaccination and booster doses as independent variables. The second part consisted of fixing the maximum and minimum values for parameters included in simulation B (all but those related to virus variants) and estimating both the thresholds obtained and the Spearman correlation coefficients with the results of simulation B. We presented the range of thresholds and correlations obtained.

The adapted compartmental model was constructed, the simulations were run, and the data were analyzed using the statistical computing and graphics software R (version 4.0.2; R Foundation for Statistical Computing) in the integrated development environment RStudio (version 2022.07.1+554; Posit). Packages *zoo* (version 1.8-10), *pROC* (version 1.18.0), and *unbalanced* (version 2.1) were used. The 95% CIs were calculated for all punctual estimates.

**Scenarios for different q estimates.**

- The *q* estimate derived from the constructed models represents the ratio between the daily expected number of cases arising from quarantined high-risk contacts and the total number of daily cases. Several parameterizations might lead to different numbers of expected cases resulting from applying the modeled transitions to the real number of quarantined individuals.
- Simplifying the presented model to a single-day transition (all quarantined individuals must progress to being infected or return to being susceptible or protected), if on any given day 100 individuals were in compartment Q and the total number of new cases was 20 and…:
  ...the infection risk (IR) was defined as 10%, the q estimate would be 0.5 (10% × 100/20). In other words, we would expect half the cases to come from quarantined individuals.
  ...the IR was defined as 20%, the q estimate would be 1.0 (20% × 100/20). In other words, we would expect all cases to come from quarantined individuals.
  ...the IR was defined as 30%, the q estimate would be 1.5 (30% × 100/20). In other words, we would expect more cases from quarantined individuals than the total number of new cases.
- Therefore, the *q* estimate determined by this model is not a proportion as *q* estimates of >1 are possible within the constructed model (see the Discussion section).

### Ethical Considerations

Our study used publicly available aggregated secondary data with no characteristics that allowed for individual identification. Therefore, the research team considered that there were no relevant data protection and privacy issues to report.

## Results

### Descriptive Analysis of the Parameterizations

The characteristics of the parameterizations used in the model and for each simulation are described in Table 3. The median of the maximum transition periods (Δtγ_max_/∆tκς_max_) was 8 days (IQR 6 for the γ transition and IQR 7 for the κ and ς transitions) and 4 days for the average transition periods (∆tγ_mean_/∆tκς_mean_; IQR 4). The main difference between the simulations is in the IR, with an average of 28.9% (SD 13.6%) verified in simulation A, which is much higher than that of 0.8% (SD 0.5%) for simulation B.

**Table 3:** Descriptive analysis of simulation parameters.

		Simulation A	Simulation B
**Variants**			
	Δtγ_max_^a^ (d), median (IQR)	8 (6)	8 (6)
	Δtγ_mean_^b^ (d), median (IQR)	4 (4)	4 (4)
	∆tκς_max_^c^ (d), median (IQR)	8 (7)	9 (7)
	∆tκς_mean_^d^ (d), median (IQR)	4 (4)	4 (4)
	IR^e^ (%), mean (SD)	28.9 (13.6)	0.8 (0.5)
**Complete vaccination (%), mean (SD)**			
	Ve_0_^f^	33.7 (16.3)	33.7 (16.3)
	Ve_max_^g^	64.8 (21.8)	64.8 (21.8)
	Ve_waned_^h^	49.2 (14.9)	49.2 (14.9)
**Booster doses (%), mean (SD)**			
	Be_0_^i^	65.1 (14.0)	65.1 (14.0)
	Be_max_^j^	80.1 (12.3)	80.1 (12.3)
	Be_waned_^k^	49.2 (14.9)	49.2 (14.9)

^a^Δtγ_max_: maximum time until γ.

^b^Δtγ_mean_: average time until γ.

^c^∆tκς_max_: maximum time until κ and ς.

^d^∆tκς_mean_: average time until κ and ς.

^e^IR: infection risk.

^f^Ve_0_: vaccine efficacy.

^g^Ve_max_: maximum vaccine efficacy.

^h^Ve_waned_: waned vaccine efficacy.

^i^Be_0_: booster dose efficacy.

^j^Be_max_: maximum booster dose efficacy.

^k^Be_waned_: waned booster dose efficacy.

### Descriptive Analysis of the Simulations

**Figure 2:**
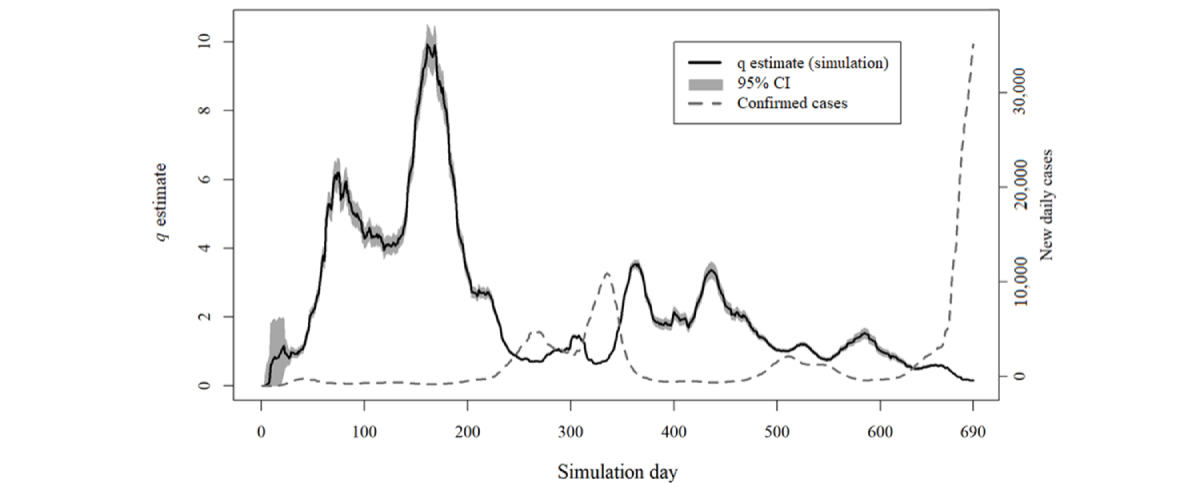
Results of simulation A in comparison with the daily total number of new COVID-19 cases at scale.

**Figure 3:**
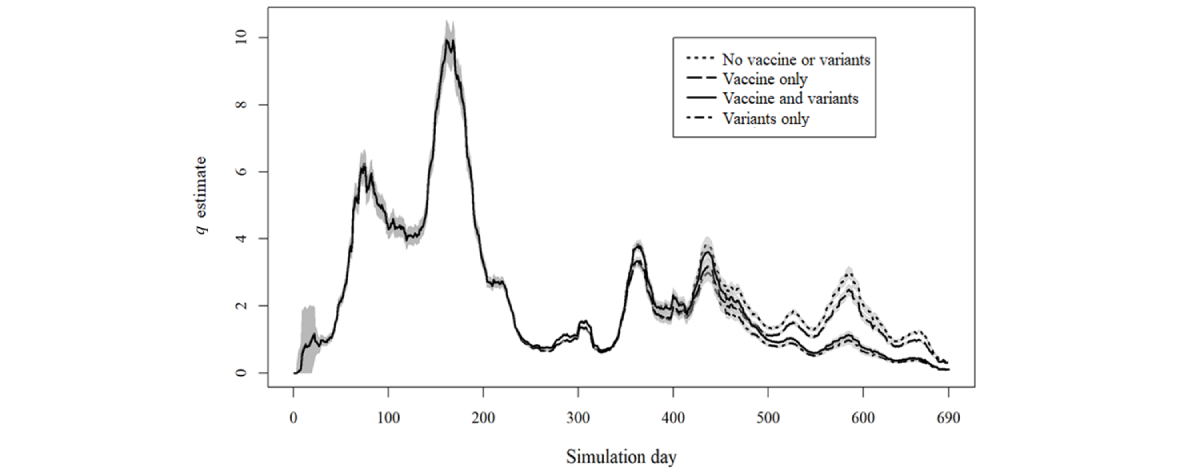
Results of simulation A separated according to inclusion or exclusion of vaccines or SARS-CoV-2 variants.

**Figure 4:**
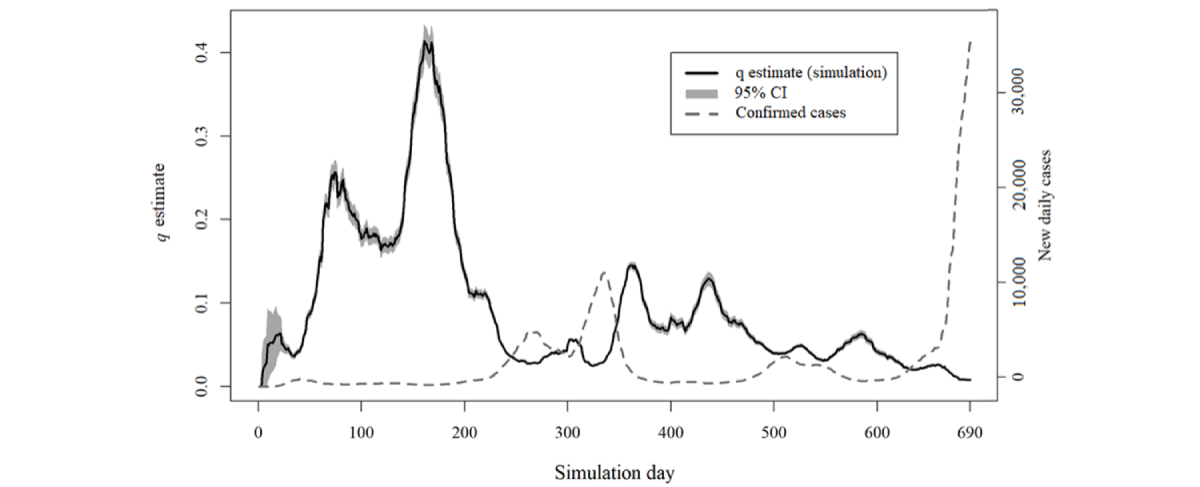
Results of simulation B in comparison with the daily total number of new COVID-19 cases at scale.

**Figure 5:**
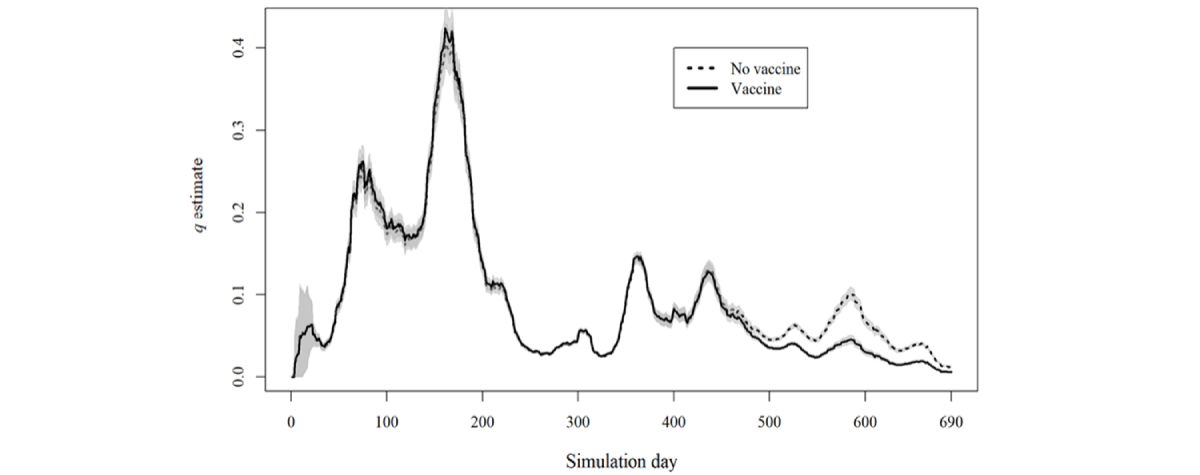
Results of simulation B separated according to the inclusion or exclusion of vaccines.

The daily value of the *q* estimate is plotted in Figures 2-5 for simulations A and B. This estimate exceeded the value of 1 at some moments in simulation A, indicating that, according to the model’s parameters, it would be expected that more infection cases resulting from quarantined individuals had occurred than the total number of new cases of COVID-19 reported for those specific days (Textbox 2).

An inverse relationship between the values of the *q* estimate and the total number of new cases of COVID-19 is ascertainable through analysis of Figures 2 and 4. Indeed, simulation A presented a correlation of −0.71 (95% CI −0.74 to −0.67), and simulation B showed a correlation of −0.76 (95% CI −0.79 to −0.73).

Despite the different procedures underlying the 2 simulations (A and B), the maximum *q* estimate in both simulations was reached on day 161. After that, the same *q* estimate never reached >50% of that maximum value.

The analysis of all iteration results according to the presence or absence of different variants and vaccination’s protective effect (Figures 3 and 5) shows the considerable overlap between the curves in either simulation A or B.

### Defining the Effectiveness Threshold

**Figure 6:**
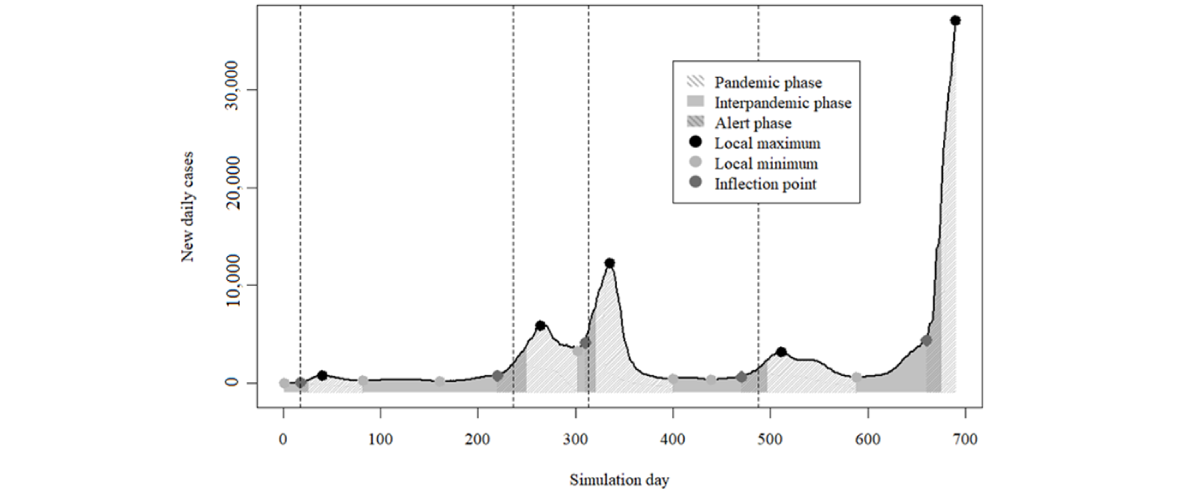
Daily COVID-19 case number evolution with identification of wave peaks and periods included in the effectiveness threshold for contact tracing. Vertical dotted lines represent the imposing of population lockdown measures by Portuguese authorities in each pandemic phase during the period of analysis.

Figure 6 shows the distribution of the number of daily cases throughout the 690 days included in the compartmental model, including the local maxima; local minima; inflection points; and pandemic, alert, and interpandemic phases. Case peaks occurred on days 40, 264, 335, and 511 of the study period. We also considered that, on day 690 (end of the study period), we were at a new peak because of the number of cases. The inflection points for each of the 5 peaks identified show that alert phases occurred between days 18 and 26 for the first pandemic phase, between days 220 and 250 for the second pandemic phase, between days 310 and 321 for the third pandemic phase, between days 470 and 497 for the fourth pandemic phase, and between days 660 and 676 for the fifth and final pandemic phase. The period included in calculating the effectiveness threshold encompassed 403 days (between days 1 and 26, days 82 and 250, days 302 and 321, days 400 and 497, and days 588 and 676 in accordance with each of the 5 pandemic phases). Table 4 shows the testing periods of the theoretical thresholds derived from both models and the classification of those periods in the alert (test-positive) and interpandemic (test-negative) phases.

For simulation A, we observed that the value of the *q* estimate that would most likely define the theoretical effectiveness threshold of contact tracing would be 1.93, with 85.7% (258/301; 95% CI 82.1%-89.7%) of alert phase days in which the *q* estimate was below the effectiveness threshold (ie, sensitivity), 66.1% (199/301; 95% CI 60.5%-71.1%) of interpandemic phase days in which the *q* estimate was above the effectiveness threshold (ie, specificity), and a proportion of alert days among days with the *q* estimate below the effectiveness threshold (ie, PPV) of 71.7% (258/360; 95% CI 68.4%-75.1%). For simulation B, the hypothetical effectiveness threshold was 0.07, with 87% (262/301; 95% CI 83.4%-90.7%) sensitivity, 66.8% (201/301; 95% CI 61.5%-72.1%) specificity, and a 72.5% (262/362; 95% CI 69.2%-75.9%) PPV.

In Portugal, population lockdowns were imposed during all pandemic phases except the fifth one [36]. In the model days, lockdowns occurred on days 18, 236, 313, and 488. Both simulations would, by default, start below the effectiveness threshold from day 1 in the model until the first pandemic phase. Unsurprisingly, by day 18, both would still stay below the effectiveness threshold. Neither of the simulations’ *q* estimates went above threshold levels between the second and third pandemic phases, which may indicate that this hypothetical estimate could be used as an argument against lifting lockdown measures, thus avoiding an interpandemic phase of just 8 days. Both models kept *q* estimates below the effectiveness threshold after the fourth pandemic phase. For the 2 remaining pandemic phases (second and fourth), simulation A broke the threshold within the respective alert phases 7 and 17 days before the actual lockdowns (days 229 and 471), and simulation B broke the threshold 4 and 16 days before the actual lockdowns (days 232 and 472).

**Table 4:** Testing periods for theoretical effectiveness threshold.

Days	Phase	Classification
1-17	Interpandemic	Negative
18-26	Alert	Positive
82-219	Interpandemic	Negative
220-250	Alert	Positive
302-309	Interpandemic	Negative
310-321	Alert	Positive
400-469	Interpandemic	Negative
470-497	Alert	Positive
588-659	Interpandemic	Negative
660-676	Alert	Positive

### Sensitivity Analysis

Tables 5 and 6 show the results of the sensitivity analysis. On the basis of the multiple linear regression results (Table 5), a 10% increase in IR for each variant was associated with an increase in the estimated threshold value of at least 0.01. The influence was maximal for the variant “other” (β=.52; *P*<.001). The influence on the result of the model for the remaining variant parameters was inconsistent, with some parameters leading to an increase in the hypothetical threshold for a certain variant and the same parameter leading to a decrease in the estimated threshold for other variants. Of these parameters, maximal and mean times until κ and ς for variant “other” were negatively related to the estimated threshold (β=−.10 and *P*<.001, and β=−.08 and *P*=.02, respectively), as were the mean times until κ and ς for the alpha and delta variants (β=−.08 and *P*=.04, and β=−.08 and *P*=.048, respectively). In contrast, an increase in the mean time until κ and ς for the omicron variant was related to an increased threshold (β=.08; *P*=.02). Regarding vaccination, only an increase in efficacy at inoculation for booster doses was negatively related to the estimated threshold (β=−.11; *P*=.02). All other vaccine efficacies did not present a significant relationship with the threshold attained.

Regarding the correlation and threshold analyses (Table 6), we obtained a minimum correlation coefficient of 0.87 and a maximum correlation coefficient of 0.99. Threshold values were highly variable except for those resulting from IR and when vaccine efficacy at inoculation (Ve_0_) was set to 60%, where the ratio between the maximum and minimum thresholds was <20. This result corroborates the results obtained in the multiple linear regression regarding the impact of IR on the threshold estimates and, consequently, on *q* estimates.

**Table 5:** Multiple linear regression model parameter coefficients for threshold determination.

Variant and model parameter			Coefficient (95% CI)		*P* value
**Other variant**					
	Δtγ_max_^a^	0.02 (−0.04 to 0.08)		.51	
	Δtγ_mean_^b^	−0.08 (−0.16 to 0.01)		.07	
	∆tκς_max_^c^	−0.10 (−0.16 to −0.05)		<.001	
	∆tκς_mean_^d^	−0.08 (−0.15 to −0.02)		.02	
	IR^e^	0.52 (0.41 to 0.64)		<.001	
**Alpha**					
	Δtγ_max_	−0.03 (−0.09 to 0.03)		.35	
	∆tγ_mean_	−0.04 (−0.12 to 0.03)		.25	
	∆tκς_max_	0.03 (−0.02 to 0.09)		.26	
	∆tκς_mean_	−0.08 (−0.15 to 0.00)		.04	
	IR	0.05 (−0.07 to 0.18)		.41	
**Beta**					
	Δtγ_max_	0 (−0.07 to 0.06)		.99	
	Δtγ_mean_	0.01 (−0.07 to 0.09)		.79	
	∆tκς_max_	0.03 (−0.04 to 0.10)		.41	
	∆tκς_mean_	0.01 (−0.07 to 0.09)		.78	
	IR	0.01 (−0.11 to 0.13)		.84	
**Gamma**					
	Δtγ_max_	−0.04 (−0.11 to 0.04)		.31	
	Δtγ_mean_	0.07 (−0.01 to 0.16)		.08	
	∆tκς_max_	0.03 (−0.02 to 0.09)		.23	
	∆tκς_mean_	0.01 (−0.06 to 0.09)		.75	
	IR	0.15 (0.03 to 0.28)		.02	
**Delta**					
	Δtγ_max_	0.04 (−0.02 to 0.10)		.19	
	Δtγ_mean_	−0.04 (−0.12 to 0.04)		.32	
	∆tκς_max_	0.02 (−0.04 to 0.09)		.47	
	∆tκς_mean_	−0.08 (−0.16 to 0.00)		.048	
	IR	0.11 (−0.04 to 0.26)		.16	
**Omicron**					
	Δtγ_max_	0.02 (−0.05 to 0.08)		.59	
	Δtγ_mean_	0.00 (−0.07 to 0.06)		.89	
	∆tκς_max_	−0.04 (−0.09 to 0.01)		.16	
	∆tκς_mean_	0.08 (0.01 to 0.15)		.02	
	IR	0.08 (−0.03 to 0.20)		.16	
Ve_0_^f^			0.02 (−0.04 to 0.09)		.48
Ve_max_^g^			0.01 (−0.05 to 0.06)		.85
Ve_waned_^h^			N/A^i^		N/A
Be_0_^j^			−0.11 (−0.20 to −0.02)		.02
Be_max_^k^			0.01 (−0.09 to 0.11)		.84
Be_waned_^l^			N/A		N/A

^a^Δtγ_max_: maximum time until γ.

^b^Δtγ_mean_: average time until γ.

^c^∆tκς_max_: maximum time until κ and ς.

^d^∆tκς_mean_: average time until κ and ς.

^e^IR: infection risk.

^f^Ve_0_: vaccine efficacy.

^g^Ve_max_: maximum vaccine efficacy.

^h^Ve_waned_: waned vaccine efficacy.

^i^N/A: not applicable; coefficients for waned efficacy were not calculated as this parameter was input in the model as the mean of the inoculation and maximum efficacies (Table 1).

^j^Be_0_: booster dose efficacy.

^k^Be_max_: maximum booster dose efficacy.

^l^Be_waned_: waned booster dose efficacy.

**Table 6:** Correlation and threshold estimates for fixed parameters.

Variable and value			Correlation^a^ (range)		Threshold (range)
**Δtγ _max_^b^ (d)**					
	2	0.87-0.88		0.003-13.372	
	14	0.88-0.99		0.002-11.057	
∆**tγ_mean_^c^ (d)**					
	2	0.92-0.98		0.037-2.965	
	14	0.89-0.95		0.002-2.696	
∆**tκς_max_^d^(d)**					
	2	0.91-0.99		0.016-13.372	
	14	0.88-0.99		0.002-11.278	
∆**tκς_mean_^e^ (d)**					
	2	0.95-0.99		0.030-7.385	
	14	0.88-0.99		0.002-3.137	
**IR^f^**					
	0.001	0.87-0.99		0.002-0.031	
	0.500	0.88-0.99		1.248-13.372	
**Ve_0_^g^**					
	0.10	0.87-0.99		0.002-13.372	
	0.60	0.90-0.96		0.046-0.448	
**Ve_max_^h^**					
	0.10	0.89-0.99		0.002-13.372	
	0.95	0.87-0.96		0.002-10.744	
**Be_0_^i^**					
	0.10	0.87-0.99		0.002-13.372	
	0.80	0.89-0.99		0.002-13.144	
**Be_max_^j^**					
	0.10	0.89-0.99		0.002-13.372	
	0.95	0.89-0.99		0.030-8.352	

^a^Compared with simulation B results.

^b^Δtγ_max_: maximum time until γ.

^c^Δtγ_mean_: average time until γ.

^d^∆tκς_max_: maximum time until κ and ς.

^e^∆tκς_mean_: average time until κ and ς.

^f^IR: infection risk.

^g^Ve_0_: vaccine efficacy.

^h^Ve_max_: maximum vaccine efficacy.

^i^Be_0_: booster dose efficacy.

^j^Be_max_: maximum booster dose efficacy.

## Discussion

### Principal Findings

In this study, we developed a compartmental model to describe the relationship among the official data on COVID-19 for Portugal, namely regarding the DGS definition of quarantined individuals. The main aim of this study was to estimate the daily ratio of cases that occurred in individuals in imposed quarantine through contact tracing (*q* estimate). Despite being a compartmental model, only transitions to and from compartment Q (quarantined individuals) were modeled. Daily data regarding all the other transitions and daily data on all compartments except compartments S (susceptible) and E (exposed individuals not traced) were already available and, therefore, not processed.

To the best of our knowledge, this study is the first attempt to use COVID-19 contact tracing data to define the infection dynamics of SARS-CoV-2. The main reason for this might be that the DGS was, as far as the authors are aware, the only national health authority with public data regarding individuals under quarantine systematically imposed by health authorities.

We obtained *q* estimates for 2 different simulations, both grounded on published evidence and some degree of parameter estimation. Simulation A was intended to reflect the presence of multiple strains with different infectious characteristics, whereas simulation B was intended to calibrate the IR of SARS-CoV-2 in quarantined individuals. The *q* estimates obtained by the 2 simulations were highly negatively correlated with the number of confirmed daily cases of COVID-19, which shows that a diminishing ratio of cases from quarantined individuals indirectly indicates the waning effectiveness of contact tracing. Assuming that we have proven this concept, it may indicate epidemiological contexts in which contact tracing as an epidemic combat measure would be insufficient, thus prompting the need to consider implementing complementary pandemic combat measures that result in further social restrictions, namely, general lockdowns.

To demonstrate the impact of applying this theoretical *q* estimate, we tried to establish, for each simulation, a hypothetical effectiveness threshold for contact tracing and relate it to the key moment for implementing general lockdowns, either by defining the phases of the pandemic and estimating a PPV (ie, the quotient of the number of alert phase days in which the *q* estimate was below the effectiveness threshold and the number of days in which the *q* estimate was below the effectiveness threshold) of each simulation or by comparing with actual lockdown measures in Portugal. We found a PPV of >70% for both simulations, and an anticipation of at least 4 days in the second and fourth lockdowns was the result of the theoretical simulations used for decision-making.

In addition, a sensitivity analysis of the parameters used to model compartment Q transitions showed that only the IR for any strain and the vaccine efficacy at booster dose inoculation did significantly affect the *q* estimates. Despite having a residual influence, the parameters input do not allow for a direct interpretation of the value of the *q* estimate, especially in simulation A as this simulation at times obtained values of >1. The scenarios where this situation could occur in our model were explained in Textbox 2. The main reasons for these estimates are more likely related to an overestimated IR in compartment Q and may include misclassification of low-risk exposures as high-risk exposures (eg, in schools when the first guidelines initially considered all same-class students as high-risk contacts regardless of mask or social distancing measures implemented [37]), delayed diagnosis, or different timing for testing in quarantined individuals (such as changes in guidelines for testing in the beginning, middle, or end of imposed quarantine [5,21,38]) and different isolation timing (some individuals might only be traced near the end of their period of isolation, which in turn leads to a reduced time in compartment Q not controlled by our model design).

Therefore, our study should be regarded as a demonstration of the application of an effectiveness threshold for contact tracing as a measure of pandemic control. Consequently, the *q* estimates and effectiveness thresholds derived from both simulations are hypothetical values but likely to be highly correlated with the true proportion of cases from quarantined individuals, as shown by our sensitivity analysis.

We deem it necessary to draw attention to the importance of open data and data sharing as a way to catalyze research and accelerate innovation and development that shortens the time between the detection of potentially epidemic pathogens and the development of appropriate containment and mitigation measures [39,40]. The advantages of data sharing will only be fully attained if data are reported on an everyday basis of definitions and procedures. This factor was not observed during the COVID-19 pandemic. This research team expects that this lesson, too, can be derived from this pandemic and that, consequently, definitions and procedures will be adapted in conformity [41].

### Limitations

The main limitation of our study arises from implementing a compartmental model and simulation methods to estimate transitions between compartments, potentially leading to results that only partially reflect reality as it occurred at each moment of the period analyzed. Furthermore, it is not possible to exclude the influence that the implementation of confinement measures or other measures implemented during each pandemic wave may have had on the proportion of new cases from exposed individuals. However, the impact of considering different parameterizations of variants or vaccination efficacy, demonstrated by the visual overlap between the results of the different simulations as well as the conducted sensitivity analysis, strengthens the potential robustness of the results and the model.

In addition, the parameters chosen include other limitations as they are based on data collected in studies that were run under controlled conditions [42]. Most of those studies also focused on the disease’s transmission dynamics [22], analyzing mainly those persons who developed the disease and the time that elapsed until infection. Therefore, we lacked estimates of the duration of epidemiological surveillance for exposed individuals who did not develop the infection. To mitigate this limitation, we assumed that this time would be similar to the time required for individuals to develop the disease.

Few studies have compared the risk of infection per virus variant throughout the quarantine period. Therefore, although we acknowledge that some variants might have been more transmissible than others, it did not allow us to determine whether this variable translated only to a higher IR or a lower time until the transition between exposed and infected (or any other hypotheses, for that matter). To mitigate the impact of this limitation, different values for parameters (which assumed that all variants could have variable IR and time to infection) had the potential to depict variant differences concerning the degree of contagion and infection. The residual impact of different variants, except for their respective IRs, on the simulation results may attenuate this limitation.

It is not possible to exclude the hypothesis that the effect of implementation of confinement measures (or other measures implemented during each pandemic phase) on the ratio of new cases from quarantined individuals may have led the model to present *q* estimates that were different from those expected without such measures. Thus, the definition of a value for the ratio of cases of quarantined individuals that could work as a threshold for lifting those more restrictive measures or restarting contact tracing as the primary pandemic combat strategy is beyond the scope of this study. This “lift measures threshold” could define the moment where the influence of confinement measures stopped manifesting. In our model, we assume that the contact tracing strategy is maintained throughout the simulation period and only use data from the interpandemic and alert phases to define the hypothetical effectiveness threshold.

Finally, given the format in which the data were collected, the analysis could be conducted only at the national level. This lack of data granularity demanded an implicit assumption that any change in the number of cases or individuals under quarantine imposed by health authorities affects national, regional, and local levels simultaneously and proportionally. Greater data detail, both geographical and sectorial, would potentially allow for determining effectiveness thresholds—and the consequent implementation of pandemic containment measures—at those differentiated levels, with a more apparent effort-benefit ratio that could be better understood and better accepted by the population. This would most likely result in higher population adherence to containment measures and finer-tuned control of both the sanitary and socioeconomic impacts of the pandemic.

### Future Work

As we have stated, this work is a starting point for using data on high-risk contacts of COVID-19 cases to define transmission dynamics of SARS-CoV-2, which could lead to further studies addressing the aforementioned limitations and discussions of alternative hypotheses and perspectives left unexplored in this study. An immediate suggestion could be the case of a scenario in which the variation in IR for compartment Q occurs within each iteration. Such dynamic IR should reflect the implementation of pandemic combat measures that have been proven to change the IR (namely, large-scale testing [11], scaling up of contact tracing [1], and population lockdowns [13,14]). Caution should be taken when applying this dynamic IR to these models to tackle *q* estimates of >1. Such an approach, without accounting for other measures, would lead to a maximization of IR in quarantined individuals, which would mean that, in pandemic phases in which cases are expected to occur mostly in non–contact-traced individuals, we would contrarily have a maximization of the *q* estimate.

Another scenario to tackle in future work—perhaps also resorting to simulation methods—would be to investigate what would happen should contact tracing come to a halt and what would be the impact of fully transferring resources allocated to this public health task to other activities regarding epidemiological dynamics and the pandemic’s impact on a country’s sanitary and economic dimensions.

As mentioned previously, the theoretical definition of contact tracing effectiveness thresholds that would allow for the relief of restrictive measures is beyond the scope of this work. Nonetheless, it might be equally valuable to define thresholds for restrictive measure relief as it could be helpful for communication by public officials during pandemic scenarios as well as to balance the scales between the implicit health-economy dichotomy that so often arose in narratives with global reach. There is also a relevant economic component to this work as it is intertwined with the efficiency of these pandemic control measures and their direct and indirect impacts on countries’ health and economy. This work might be very helpful in drawing lessons that better enable us to adapt our society to events such as pandemics.

Finally, and from a health economics standpoint, it would be interesting to understand how the population perceives contact tracing as an instrument used by health authorities to contain pandemics and what the perception would be, for example, in terms of adherence to pandemic control measures should contact tracing cease.

### Conclusions

Our work provides important information for policy and decision makers, namely in terms of epidemiology and pandemic/crisis management, as well as for public health professionals. It also constitutes a relevant source of information and an objective acknowledgment of the importance of contact tracing, which was widely used worldwide in all stages of the COVID-19 pandemic.

Concretely, this study, although using secondary data, allowed us to (1) provide a proof concept for using estimates of an effectiveness threshold for contact tracing as a primary measure of pandemic containment and (2) consider the potential use of this effectiveness threshold as a decision variable for imposing more restrictive measures, namely, lockdowns, along with indicators that were used to assess the pandemic situation (such as the transmissibility indexes and case incidence). Our results are consistent with this last possibility and, notwithstanding the presented limitations, reveal a path that, we hope, deserves further exploration.

## Abbreviations

DGS: Directorate-General of Health
DSSG: Data Science for Social Good
IR: infection risk
PPV: positive predictive value
SEIR: susceptible-exposed-infected-recovered

## Data Availability

The data sets generated and analyzed during this study are available from the corresponding author upon reasonable request. The code we developed and on which these simulations are based is available on a GitHub repository [43].
